# HbA1C Variability as an Independent Predictor of Diabetic Retinopathy: A 10-Year Retrospective Nested Case-Control Study in a Middle Eastern Population

**DOI:** 10.7759/cureus.116597

**Published:** 2026-09-20

**Authors:** Dima Abdelmannan, Heithem Ajlouni, Marwan Zidan, Manal Albuflasa, Olga Ceron, A. Enrique Caballero

**Affiliations:** 1 Diabetes and Endocrinology, Dubai Health, Dubai, ARE; 2 Research and Graduate Studies, Dubai Health, Dubai, ARE; 3 Ophthalmology, Joslin Diabetes Center, Boston, USA; 4 Division of Endocrinology, Diabetes and Metabolism, Mass General Brigham, Harvard Medical School, Boston, USA

**Keywords:** diabetes type 2, hba1c variability, microvascular complications, microvascular complications of diabetes, retinopathy, type 2 diabetes mellitus (dm), variability

## Abstract

Background

Diabetic retinopathy (DR) is a leading cause of vision impairment worldwide and a major microvascular complication of type 2 diabetes mellitus. Although chronic hyperglycemia is a well-established risk factor, evidence is accumulating that long-term glycemic variability may independently contribute to DR development. Data from Middle Eastern and Gulf Cooperation Council populations remain scarce.

Methods

We conducted a retrospective case-control study nested within a 10-year diabetes cohort at the Dubai Diabetes Center, a specialized tertiary facility in the United Arab Emirates. Cases (n = 67) who developed DR during follow-up were matched 1:1 by age to DR-free controls (n = 67). Long-term glycemic variability was assessed using the standard deviation (SD) and coefficient of variation (CV) of serial HbA1c measurements collected twice yearly. Five multiple logistic regression models evaluated the independent association between HbA1c variability and DR, adjusting for diabetes duration, baseline HbA1c, neuropathy, smoking, and insulin use.

Findings

Patients who developed DR had significantly higher HbA1c variability than controls (median SD 1.00% vs 0.55%, p < 0.001; median CV 12.34% vs. 7.93%, p < 0.001). In multivariate analyses, HbA1c variability remained independently associated with DR across all five models, whether expressed as SD (adjusted OR up to 13.85 per 1% increase) or CV (adjusted OR 1.20 per 1% increase), after adjustment for follow-up mean HbA1c. Neuropathy, smoking, and insulin use were also independently associated with DR. ROC analysis showed acceptable discriminatory performance for both SD (AUC 0.746) and CV (AUC 0.716).

Interpretation

Long-term HbA1c variability was independently associated with DR in this Middle Eastern cohort, beyond mean glycemic control. These findings suggest that serial HbA1c measurements - already collected in routine diabetes care - may provide a scalable, low-cost tool for early DR risk stratification. Prospective validation in larger, diverse populations is needed before these findings can be incorporated into clinical algorithms.

## Introduction

Diabetic retinopathy (DR) is one of the most serious microvascular complications of diabetes mellitus (DM) and remains a leading cause of vision impairment and blindness globally. It is a major cause of vision loss among working-age adults, with a substantial impact on individuals with diabetes [1]. The progression of DR can be insidious, and many patients remain asymptomatic until the disease is advanced, limiting opportunities for early intervention.

The prevalence of DR varies substantially across populations, influenced by demographic and clinical factors. Regional data show DR prevalence rates of approximately 14·4% in Oman [2] and 19% in the United Arab Emirates (UAE) [3], with substantial variation in prevalence estimates reported across studies from Saudi Arabia [4]. As the incidence of diabetes continues to rise throughout the Middle East, the associated complications, including DR, nephropathy, and neuropathy, pose an increasing public health burden.

The pathogenesis of DR is multifactorial. Established risk factors include chronic hyperglycemia, hypertension, dyslipidemia, and longer duration of diabetes [5]. Poor glycemic control, most often assessed by glycated hemoglobin (HbA1c), is a key determinant of DR development and progression. Evidence regarding the additional effect of glycemic variability has historically been less consistent; an analysis in type 1 diabetes, for example, did not demonstrate an independent association between glucose variability and retinopathy or nephropathy after accounting for mean glycemic exposure [6]. Large epidemiologic and longitudinal studies have also emphasized the importance of glycemic exposure, diabetes duration, and blood pressure in determining DR risk [7,8].

In recent years, attention has increasingly shifted toward long-term glycemic variability, including visit-to-visit fluctuations in HbA1c. Observational studies, clinical-trial analyses, and reviews have associated HbA1c variability with adverse diabetes outcomes, with several studies specifically demonstrating an association with DR [9-12]. Proposed mechanisms include increased oxidative stress, endothelial dysfunction, and inflammatory signaling, although the independent causal contribution of glycemic variability remains incompletely established [11]. HbA1c variability offers a practical measure of long-term glycemic instability because it can be derived from routinely collected clinical measurements. Evidence specifically examining its relationship with DR remains limited in Gulf populations, providing the rationale for the present study.

Conducted at the Dubai Diabetes Center, a multidisciplinary clinic serving a predominantly Emirati population, this study leverages a decade of follow-up data to evaluate whether long-term HbA1c variability is independently associated with incident DR and to identify other clinical predictors of DR risk. The patient population, largely homogeneous in demographic background, provides a unique opportunity to evaluate emerging biomarkers, including HbA1c variability, in a real-world clinical setting.

The primary objective of this study was to determine whether long-term HbA1c variability, measured by standard deviation (SD) and coefficient of variation (CV), is independently associated with incident DR after adjustment for mean HbA1c and established clinical risk factors. Secondary objectives were to compare the discriminatory performance of SD and CV using ROC analysis and to identify other independent clinical predictors of DR in this Middle Eastern cohort. A retrospective nested case-control design was used to achieve these objectives.

## Materials and methods

Study design and participants

This was a retrospective case-control study conducted at the Dubai Diabetes Center, a specialized tertiary facility for adults with type 2 diabetes mellitus (T2DM). A total of 67 patients who developed DR during a 10-year follow-up period were identified. These patients were matched by age to a control group of 67 patients who remained free of DR throughout the same follow-up duration. Sex was not used as a matching criterion but was compared between groups and included as a covariate in sensitivity analyses.

The total final sample included for analysis consisted of a total of 134 patients for analysis. DR diagnosis was confirmed through retinal imaging, and the DR severity was classified according to the International Clinical Diabetic Retinopathy Disease Severity Scale [13]. Baseline was defined as the date of the first clinic visit between 2009 and 2013.

Fundus photography was performed at baseline and at each annual clinic visit over the 10-year follow-up period or sooner if the patient reported visual symptoms. Retinal images were graded for the presence and severity of DR by a masked qualified ophthalmologist using the International Clinical Diabetic Retinopathy Disease Severity Scale [13].

Ethical consideration

This study was conducted in accordance with the ethical principles of the Declaration of Helsinki. Ethical approval was obtained from the Dubai Scientific Research Ethics Committee (DSREC) at Mohammed Bin Rashid University of Medicine and Health Sciences (approval number: MBRU IRB-2025-150). All patient data were fully de-identified prior to analysis, and confidentiality was maintained throughout the study. Informed consent was waived by the ethics committee given the retrospective nature of the study, and no identifiable patient information was accessed or used.

Data collection

The following data were extracted from patient records across the 10-year follow-up period:

Demographics: Age and gender (ethnicity was not recorded, though >90% of the patient population was Emirati).

Clinical variables: Duration of diabetes (years), smoking status (never, former, current), hypertension, dyslipidemia, neuropathy, nephropathy (assessed via albumin-to-creatinine ratio, ACR), and coronary artery disease.

Glycemic and metabolic parameters: Serial HbA1c values (collected twice per year), body mass index (BMI), systolic and diastolic blood pressure, lipid profile (total cholesterol, HDL, LDL, triglycerides), and estimated glomerular filtration rate (eGFR).

Medications: Diabetes-related medications were categorized as oral hypoglycemic agents, insulin (monotherapy or combination), and GLP-1 receptor agonists. Cardiovascular medications were also recorded.

Retinopathy classification: DR status was classified using the International Clinical Diabetic Retinopathy Disease Severity Scale (Wilkinson et al., 2003), with stages including no DR, mild/moderate/severe non-proliferative diabetic retinopathy (NPDR), and proliferative diabetic retinopathy (PDR). When available, diabetic macular edema (DME) was also recorded and classified as center-involving or non-center-involving, though DME was not formally analyzed.

HbA1c variability calculation

Glycemic variability refers to fluctuations in blood glucose levels over time and can be assessed through various short- and long-term measures. In this study, we evaluated long-term glycemic variability using serial HbA1c measurements, which serve as a clinically accessible and validated proxy for chronic glucose fluctuation.

For each participant, HbA1c variability was quantified using the following statistical measures: Standard deviation (SD) represents the absolute magnitude of HbA1c fluctuation over time. Coefficient of variation (CV) reflects the relative variability and was calculated as CV (%) = (SD / Mean HbA1c) × 100.

These metrics were calculated using all available HbA1c values collected over the follow-up period.

For participants who developed DR, HbA1c variability was measured from the baseline visit up to the last recorded HbA1c result prior to the confirmed diagnosis of DR.

For participants who remained DR-free, variability was calculated from baseline through their final follow-up HbA1c measurement, ensuring comparable observation durations between groups.

Both SD and CV were analyzed as continuous measures of long-term HbA1c variability. Although thresholds for HbA1c variability have been proposed in the literature, no universally accepted clinical cutoff has been established [11]; therefore, no fixed SD or CV threshold was used in the primary analyses.

Peripheral neuropathy was defined by clinical examination findings (vibration threshold and monofilament testing). Nephropathy was defined by albumin-to-creatinine ratio thresholds per KDIGO guidelines (microalbuminuria: ACR 30-300 mg/g; macroalbuminuria: ACR >300 mg/g).

Inclusion and exclusion criteria

Patients were eligible if they had (1) confirmed T2DM, (2) age ≥18 years, (3) an initial clinic visit at the Dubai Diabetes Center between 2009 and 2013, (4) a minimum follow-up of 10 years, and (5) at least five or more serial HbA1c measurements during follow-up. Cases in the DR group were required to have DR-free status confirmed at baseline by fundus photography. Patients were excluded if they had type 1 diabetes mellitus, pre-existing DR at baseline, fewer than five HbA1c measurements, missing or ungraded baseline retinal imaging, other identifiable non-diabetic causes of retinopathy, or incomplete medical records precluding extraction of key variables.

Sample size calculation

Sample size was estimated based on the ability to detect differences in HbA1c variability between groups. For SD-based analysis, a sample of 62 per group would provide 90% power to detect a 0·3% difference in SD (assuming SDs of 0.6% vs. 0.4%) using a two-sided unequal-variance t-test. For CV-based analysis, the same sample size would detect a 3% difference in CV (assuming SDs of 6% vs. 4%). Considering the reduced power of non-parametric tests, 5% was added, yielding a final required sample size of 65 per group. The final sample included 67 patients in each group.

Statistical analysis

All statistical analyses were performed using IBM SPSS Statistics, Version 29.0 (IBM Corp., Armonk, NY, released 2022). A two-tailed p-value < 0.05 was considered statistically significant throughout. Continuous variables were summarized as means ± standard deviation (SD) for normally distributed data or medians and interquartile ranges (IQR) for non-normally distributed data. Categorical variables were expressed as counts and percentages. The differences between patients who developed DR and those who remained DR-free were assessed using independent t-tests for normally distributed continuous variables, Mann-Whitney U tests for skewed continuous variables, and Chi-square or Fisher’s exact tests for categorical variables, as appropriate. Five logistic regression models were constructed to evaluate the independent effect of HbA1c variability on DR risk: Model A: HbA1c mean + CV; Model B: HbA1c mean + SD; Model C: HbA1c mean only; Model D: SD only; Model E: CV only.

Due to collinearity between SD and CV, they were not included together in the same model. This approach allowed us to isolate and confirm the predictive value of glycemic variability, independently from mean glycemic exposure. Covariates included diabetes duration, baseline HbA1c, insulin use, neuropathy, and smoking, selected based on univariate significance (p < 0·10) and clinical relevance. To address potential multicollinearity between SD and CV, these metrics were not included simultaneously in any single model.

Missing data were handled using complete case analysis; formal multiple imputation was not performed. Patients with fewer than five serial HbA1c measurements were excluded to ensure stable variability estimates. Any remaining missing values in covariates were minimal and handled by listwise deletion.

## Results

Demographic and clinical characteristics

A total of 134 participants were included in the analysis, with 67 in the DR group and 67 in the non-DR (NDR) group. Demographic, clinical, and biochemical characteristics are summarized in Table 1.

**Table 1:** Baseline demographics and glycemic variability characteristics of participants with and without diabetic retinopathy (DR) Notes: Age and BMI are reported as mean ±SD; t-test was used for group comparison. **¹ Reported as median (interquartile range); Mann-Whitney U test was used for group comparison. † Statistically significant at p < 0.05.

Baseline characteristics	DR group (n = 67)	NDR group (n = 67)	p-value
Age (years)	47.7 ± 14.1	47.7 ± 11.6	0·995
Gender (% male)	56.7%	49.3%	0·387
Duration of diabetes (years)**¹	7 (4 –11)	5 (1 -10)	0·014†
BMI (kg/m²)	30.5 ± 5.6	31.2 ± 7.2	0·535
Baseline HbA1c (%)**¹	8.6 (7.7-9.8)	7.4 (6.5-8.3)	<0·001†
Neuropathy (%)	35.8%	10.4%	<0·001†
Smoking (current or former) (%)	53.7%	26.9%	0·002 †
Insulin use (any form) (%)	61.2%	17.9%	<0·001†
Hypertension (%)	33.3	36.4	0·715
Dyslipidemia (%)	50.0%	43.9%	0·530
Follow-up glycemic variability metrics			
Follow-up mean HbA1c (%)**¹	8 (7.2-9.0)	7 (6.6-7.5)	<0·001†
Standard deviation (SD) (%)**¹	1.00 (0.07–2.84)	0.55 (0.19–1.52)	<0·001†
Coefficient of variation (%)**¹	12.34 (0.77–30.00)	7.93 (3.35–16.55)	<0·001†

Patients in the DR group had significantly higher median HbA1c levels (8.6%, IQR: 7.7-9.8) compared to the NDR group (7.4%, IQR: 6.5-8.3; p < 0.001). The median duration of diabetes was longer in the DR group (seven years, IQR: 4.0-11.0) versus the NDR group (5.0 years, IQR: 1.0-10.0; p = 0.014). Follow-up mean HbA1c was also significantly higher in the DR group (median 8.0%, IQR: 7.2-9.0) compared to the NDR group (7.0%, IQR: 6.6-7.5; p < 0.001), consistent with sustained differences in glycemic control over the observation period (Table 1).

Neuropathy was significantly more prevalent in the DR group (35.8%) than in the NDR group (10.4%; p < 0.001), and a greater proportion were current or former smokers (53.7% vs. 26.9%; p = 0.002). Use of insulin therapy, either alone or in combination, was notably higher in the DR group (61.2% vs. 17.9%; p < 0.001).

There were no statistically significant differences between groups in age, gender, BMI, blood pressure, lipid profiles, or the prevalence of hypertension and dyslipidemia. 

HbA1c variability analysis

To evaluate long-term glycemic variability, two key metrics were calculated using all available HbA1c readings during the follow-up period. The follow-up readings were measured twice a year (every six months): 1) standard deviation (SD): quantifying absolute glycemic fluctuations; 2) coefficient of variation (CV): SD divided by mean HbA1c, expressed as a percentage.

In the DR group, variability metrics were calculated from baseline to the last HbA1c measurement prior to retinopathy diagnosis. For NDR patients, measurements spanned the entire follow-up period. This ensured comparable observation durations.

The results (Table 1) showed significantly greater glycemic variability in the DR group:

Median SD: 1.00% (range: 0.07-2.84%) in DR vs. 0.55% (range: 0.19-1.52) in the NDR group; p < 0.001

Median CV: 12.34% (range: 0.77-30%) in DR vs. 7.93% (range: 3.35-16.55%) in the NDR group; p < 0.001

Because of high collinearity, only SD or CV was included in the regression model. The distribution of SD and CV values by group is illustrated in Figure 1. These findings are consistent with HbA1c variability having potential value as a biomarker of HbA1c variability as an early biomarker for DR risk, though prospective validation is required. 

**Figure 1:**
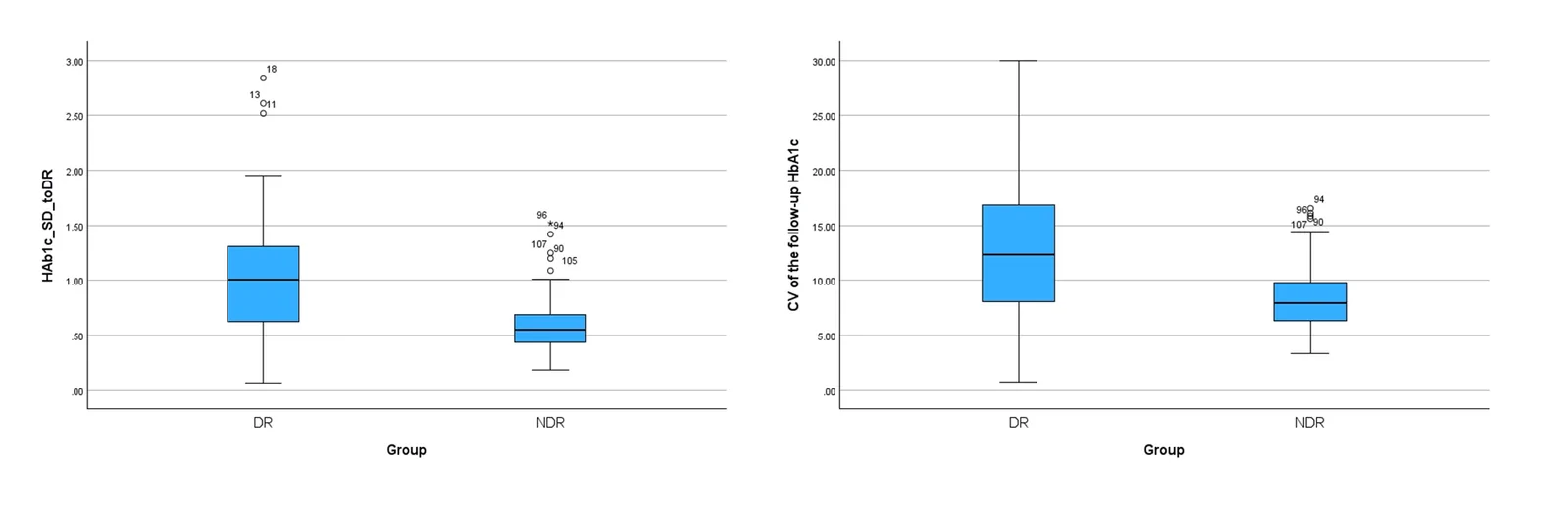
Distribution of standard deviation (SD) and coefficient of variation (CV) values by group

Multivariate logistic regression analysis

Multivariate logistic regression was performed to identify independent predictors of DR. Variables included in the model were selected based on the univariate p-value threshold of < 0.10. Tables 2-3 are different models for the logistic regression to investigate the independent risk factors of developing DR. Each model included the following five significant factors in the univariate analysis; the duration of the diabetes at the baseline, the HbA1c at the baseline, neuropathy, smoking, and using any form of insulin in addition to the follow-up HbA1c mean and the follow-up HbA1c CV (model A), the follow-up HbA1c mean and the follow-up HbA1c SD (model B), the follow-up HbA1c mean only (model C), or follow-up HbA1c SD only (model D), or the follow-up HbA1c CV only (model E). These different models allow us to test the effect of the variability of the follow-up HbA1c measured by the SD alone, the CV alone, or after adjusting for the follow-up HbA1c mean. In all the models, the variability of the follow-up HbA1c (whether as SD or CV) remains independently associated with DR whether adjusting for the mean follow-up readings or not.

**Table 2:** Multiple logistic regression models (A, B, and C) to investigate the independent risk factors of DR including the follow-up HbA1c mean

Variable	Model A		Model B		Model C	
	Adj. OR (95% CI)	p-value	Adj. OR (95% CI)	p-value	Adj. OR (95% CI)	p-value
Duration of diabetes (per year)	1.05 (0.98–1.12)	0.209	1.05 (0.98–1.12)	0.201	1.04 (0.98–1.12)	0·210
Neuropathy (yes)	5.26 (1.4–19.7)	0.014	5.23 (1.41–19.47)	0.014	5.09 (1.6–16.16)	0·006
Baseline HbA1c (%)	0.64 (0.41–0.99)	0.046	0.65 (0.42–1.0)	0.051	0.83 (0.58–1.2)	0·321
Smoking (current/former)	3.22 (1.18–8.75)	0.022	3.12 (1.16–8.42)	0.024	2.24 (0.92–5.47)	0·077
Insulin use (yes)	3.95 (1.4-11.17)	0.010	3.85 (1.37–10.80)	0.010	4.48 (1.74–11.47)	0·002
Follow-up mean HbA1c (%)	3.33 (1.62–6.82)	0.001	2.69 (1.33–5.43)	0.006	3.04 (1.57–5.90)	<0·001
Follow-up HbA1c variability (per 1% CV increase)	1.22 (1.09–1.38)	<0.001	Not included in the model		Not included in the model	
Follow-up HbA1c variability (per SD increase)	Not included in the model		12.28 (2.72–55.37)	0.001	Not included in the model	

**Table 3:** Multiple logistic regression models (D and E) to investigate the independent risk factors of diabetic retinopathy (DR) excluding the follow-up HbA1c mean.

Variable	Model D		Model E	
	Adj. OR (95% CI)	p-value	Adj. OR (95% CI)	p-value
Duration of diabetes (per year)	1.04 (0.97-1.12)	0.232	1.04 (0.97-1.12)	0·240
Neuropathy (yes)	3.76 (1.1-12.85)	0.035	3.55 (1.08-11.66)	0·037
Baseline HbA1c (%)	0.93 (0.66-1.3)	0.669	1.05 (0.77-1.43)	0·762
Smoking (current/former)	3.6 (1.39-9.32)	0.008	3.6 (1.41-9.19)	0·007
Insulin use (yes)	4.14 (1.53-11.20)	0.005	4.2 (1.58-11.19)	0·004
Follow-up mean HbA1c (%)	Not included in the model		Not included in the model	
Follow-up HbA1c variability (per 1% CV increase)	Not included in the model		1.2 (1.07-1.34)	0·001
Follow-up HbA1c variability (per SD increase)	13.85 (3.26-58.79)	<0.001	Not included in the model	

After adjusting for the HbA1c follow-up readings, the duration of diabetes and the baseline HbA1c are no longer independent risk factors, while neuropathy and insulin use remain independent risk factors in all the models. Smoking reached statistical significance in Models A, B, D, and E, but not C (p = 0.077). There was no substantial change in the adjusted OR for smoking and insulin use between the different models, while there was a decrease in the adjusted OR for neuropathy when we adjusted for the variability of HbA1c readings alone (models D and E).

Model B shows that whenever the follow-up HbA1c mean increases by one unit (1%), the odds of DR increase to 2.69-folds given that the SD of the follow-up HbA1c and the other factors are the same. Also, it shows that whenever the SD of the follow-up HbA1c increases by one unit (the same unit as the HbA1c (1%)), the odds of DR increase to 12.28-fold given that the mean of the follow-up HbA1c and the other factors in the model are the same (Table 2). Model D shows that, whenever the SD of HbA1c follow-up readings increases by one unit (1%), the odds of DR increase 13.85-fold, regardless of the mean of the follow-up readings of HbA1c, given that the other factors in the model are the same.

## Discussion

This study identified key clinical, metabolic, and treatment-related predictors of DR over a 10-year period in a Middle Eastern diabetes cohort. Our findings reinforce the role of both traditional risk factors and long-term glycemic variability in DR pathogenesis, expanding the understanding of microvascular risk.

Among classical risk factors, longer diabetes duration was significantly associated with DR on univariate analysis (p = 0.014), consistent with prior studies linking prolonged hyperglycemic exposure to cumulative microvascular damage [5]. However, after adjustment for follow-up HbA1c and variability metrics in the multivariate models, diabetes duration was no longer an independent predictor (p = 0.201-0.240 across all five models), suggesting that its effect is mediated through glycemic control over time rather than duration per se. Similarly, albuminuria, assessed via albumin-to-creatinine ratio (ACR), was significantly higher in the DR group. This supports the known association between nephropathy and DR, likely mediated through shared pathways involving endothelial dysfunction, inflammation, and vascular permeability [4,7]. Albuminuria has been proposed as a marker of systemic microvascular stress and may reflect a general predisposition to vascular injury in diabetes.

Peripheral neuropathy was also significantly more prevalent among patients with DR. Co-occurrence of diabetic microvascular complications, including retinopathy, nephropathy, and neuropathy, has been observed in clinical populations [2]. Smoking also remained independently associated with DR in most of our multivariable models. However, the published relationship between smoking and DR is heterogeneous. A meta-analysis found an increased risk of DR among smokers with type 1 diabetes but did not demonstrate a positive association in patients with type 2 diabetes [14]. Therefore, the association observed in our cohort should be interpreted as a cohort-specific finding that may also be influenced by residual confounding.

While chronic hyperglycemia is a known driver of microvascular complications, our findings reinforce that HbA1c variability independently contributes to DR risk, beyond mean glycemic levels. This supports the concept that glycemic instability may promote retinal injury through oxidative stress, mitochondrial dysfunction, and vascular inflammation. These findings align with growing evidence suggesting that long-term glycemic variability may provide information about DR risk beyond mean glycemic exposure [9,11,12].

To evaluate the independent contribution of glycemic variability, we used five multivariate logistic regression models incorporating SD and CV both independently and with adjustment for mean HbA1c. Using distinct regression models allowed us to confirm that HbA1c variability, measured via SD or CV, supports the potential value of an independent predictor for DR regardless of mean HbA1c, pending prospective validation. These variability metrics in routine clinical evaluation. Of note, baseline HbA1c showed a reversed-direction OR in Models A and B (OR 0.64 and 0.65 respectively; p = 0.046 and 0.051), an unexpected finding that likely reflects a suppressor effect: when follow-up HbA1c variability and mean are simultaneously modelled alongside baseline HbA1c, the baseline value loses independent predictive power, and its coefficient reverses, consistent with conditional collinearity between baseline and follow-up glycemic measures. This pattern was not observed in Models C, D, and E, where baseline HbA1c was not significant (p = 0.321-0.762), supporting this interpretation.

Insulin use likely reflects a more advanced or poorly controlled diabetic state, and its association with DR in our models should be interpreted as a surrogate indicator of long-term glycemic burden rather than a direct biological mediator. This is the concept of confounding by indication, whereby the indication for treatment (disease severity) - rather than the treatment itself - drives the observed association.

Interestingly, BMI, lipid profile, and blood pressure were not significantly different between groups. While these are important cardiovascular risk factors, their association with DR remains inconsistent. Our results support the notion that microvascular outcomes may be more strongly linked to renal and neurologic markers than traditional cardiometabolic parameters [8].

In our study, HbA1c variability was a robust and independent predictor of DR across all multivariate models, whether modeled as SD or CV. Notably, even after adjusting for mean HbA1c during follow-up, both measures of variability remained statistically significant. This suggests that intra-individual fluctuations in glycemic control over time confer risk beyond average glycemia. The published evidence regarding long-term glycemic variability and DR has not been entirely uniform. In the RIACE study, Penno et al. found that HbA1c variability was independently associated with nephropathy but not retinopathy [15]. In contrast, Hsing et al. reported an association between glycemic variability and DR progression [16]. In the Rio de Janeiro Type 2 Diabetes Cohort Study, Cardoso et al. found that glycemic variability did not predict retinopathy development or progression in the overall cohort after adjustment, although an association was observed among patients with better mean glycemic control [17]. More recently, a meta-analysis by Sartore et al. found a significant overall association between long-term HbA1c variability and retinopathy in type 2 diabetes [18]. Against this background of heterogeneous prior findings, our results add evidence that long-term HbA1c variability may contribute information about DR risk beyond mean glycemic exposure

Mechanistically, glycemic variability may contribute to microvascular injury through oxidative stress, endothelial dysfunction, inflammatory signaling, and impaired vascular homeostasis. Reviews specifically examining glycemic variability and DR have highlighted these pathways as biologically plausible links between glucose fluctuations and retinal injury [19]. Experimental human data have also demonstrated that oscillating glucose can produce greater endothelial dysfunction and oxidative stress than sustained hyperglycemia [20]. Broader mechanistic reviews similarly implicate oxidative stress, inflammatory signaling, and endothelial injury as potential pathways linking glycemic variability with diabetic vascular complications [21]. These mechanisms provide biological plausibility for the association observed in the present study but do not establish causality.

Clinically, our findings suggest that long-term glycemic variability may provide information about DR risk beyond mean HbA1c alone. Serial HbA1c measurements can be used to calculate SD or CV, aiding in the identification of high-risk patients who may benefit from intensified monitoring or therapy adjustment. Interestingly, the odds ratio associated with SD was substantially higher (OR = 12.28 per 1% increase), possibly reflecting its sensitivity to outliers and extremes in glycemic fluctuation. Although SD demonstrated a larger effect estimate, CV expresses variability relative to the individual's mean HbA1c and may therefore facilitate comparisons across patients. Both metrics demonstrated potential value in assessing long-term glycemic variability

Although our primary analysis focused on HbA1c variability, variability in other metabolic parameters may also contribute to microvascular risk. In a post hoc analysis of the ACCORD trial, greater visit-to-visit variability in body mass index and waist circumference was associated with some diabetic microvascular complications [22]. Our study was not designed to assess variability in other cardiometabolic measures because of inconsistent measurement frequency, but future prospective studies could examine whether variability in body weight, blood pressure, and lipid parameters provides additional information for microvascular risk stratification.

Although some patients had diabetic macular edema (DME), inconsistencies in optical coherence tomography (OCT) reporting precluded formal analysis. Because DME is an important cause of vision impairment in diabetes and OCT is central to its diagnosis and assessment, future studies should incorporate standardized DME definitions and systematic OCT-based evaluation alongside DR staging [23].

In summary, HbA1c variability emerged as a strong and independent predictor of DR, even after adjusting for traditional risk factors. Patients with greater long-term glycemic fluctuations were significantly more likely to develop DR, underscoring the potential utility of variability as a clinically meaningful biomarker. In addition to glycemic measures, other independent predictors included neuropathy, insulin use, and smoking (in most models), while demographic characteristics and lipid profiles were not associated with DR in this cohort. ROC analysis further supported the predictive value of HbA1c variability, with both SD (AUC = 0.746) and CV (AUC = 0.716) demonstrating acceptable discriminatory performance.

## Conclusions

This study demonstrates that long-term HbA1c variability, assessed using the SD and CV of serial measurements, is an independent and clinically relevant predictor of DR in a Middle Eastern cohort, even after adjustment for mean glycemic control and established risk factors. Greater intra-individual HbA1c fluctuation was associated with significantly higher odds of DR development across all five logistic regression models, with acceptable discriminatory performance confirmed by ROC analysis (SD AUC 0.746; CV AUC 0.716). While SD demonstrated a larger effect size, CV may be easier to standardize across patients with differing baseline glycemic levels, and both metrics warrant further evaluation for incorporation into future DR risk-stratification approaches. On univariate analysis, diabetes duration and albuminuria were also significantly associated with DR; in multivariate models, neuropathy, insulin use, and smoking (in most models) remained independent predictors. These findings support a more nuanced approach to DR risk stratification that incorporates glycemic variability alongside traditional risk markers. Because HbA1c SD and CV are calculable from routine serial measurements already collected in standard diabetes care, they represent scalable, low-cost additions to existing risk algorithms, particularly valuable in high-burden regions. Prospective studies should evaluate whether targeted interventions to reduce glycemic variability can delay DR onset, and future work should expand variability analysis to include other cardio-metabolic parameters such as blood pressure, lipids, and body weight, to further refine microvascular risk prediction.

This study is a retrospective, single-center analysis and is subject to inherent biases, including selection bias and residual confounding. While all patients received standardized care at a specialized diabetes center, the findings may not be fully generalizable to broader populations or primary care settings. Although HbA1c variability was rigorously assessed using serial values, variability in other important metabolic markers such as blood pressure, lipid levels, and body weight could not be evaluated due to inconsistent documentation. Additionally, the sample size of 134 participants (67 cases and 67 controls) is relatively small, limiting statistical power and increasing the risk of model overfitting across five logistic regression models. The large OR estimates - particularly for HbA1c SD - should be interpreted cautiously and may reflect instability in parameter estimation rather than true population effect sizes. Furthermore, patients who attend clinic more frequently may accumulate more serial HbA1c measurements and undergo more retinal assessments, introducing potential surveillance bias that cannot be excluded in this retrospective design. Finally, complete case analysis was used throughout; if data were not missing at random, this approach may have introduced additional selection bias. These limitations underscore the need for prospective, externally validated studies in larger and more diverse populations before HbA1c variability metrics can be recommended for routine clinical risk stratification. Another limitation of this study is the absence of objective data on treatment adherence and compliance. Non-adherence to insulin and oral antidiabetic therapy is a well-recognized driver of HbA1c variability and may partially explain the observed differences between groups. Despite these limitations, the study’s long-term follow-up, use of serial glycemic data, and focus on a Middle Eastern population provide valuable insights into DR risk stratification in underrepresented settings.
