# Correction: HbA1C Variability as an Independent Predictor of Diabetic Retinopathy: A 10-Year Retrospective Nested Case-Control Study in a Middle Eastern Population

**DOI:** 10.7759/cureus.c485

**Published:** 2026-10-05

**Authors:** Dima Abdelmannan, Heithem Ajlouni, Marwan Zidan, Manal Albuflasa, Olga Ceron, A. Enrique Caballero

**Affiliations:** 1 Diabetes and Endocrinology, Dubai Health, Dubai, ARE; 2 Research and Graduate Studies, Dubai Health, Dubai, ARE; 3 Ophthalmology, Joslin Diabetes Center, Boston, USA; 4 Division of Endocrinology, Diabetes and Metabolism, Mass General Brigham, Harvard Medical School, Boston, USA

This article has been corrected to update Dr. Augusto Enrique Caballero’s affiliation to Division of Endocrinology, Diabetes and Metabolism, Mass General Brigham, Harvard Medical School, Boston, USA.

